# Effects of high summer temperatures on heatstroke-related ambulance dispatches in Japan: A nationwide time-stratified case-crossover analysis

**DOI:** 10.1016/j.pmedr.2025.103134

**Published:** 2025-06-09

**Authors:** Keita Wagatsuma

**Affiliations:** a Division of International Health (Public Health), Graduate School of Medical and Dental Sciences, Niigata University, Niigata, Japan; b Institute for Research Administration, Niigata University, Niigata, Japan

**Keywords:** Ambient temperature, Heatstroke, Ambulance dispatch, Case-crossover, Japan

## Abstract

**Objective:**

While the short-term effects of ambient temperature on heatstroke mortality have been studied across various countries, the impact on morbidity and its spatial distribution remains insufficiently examined. We quantified the association between maximum temperature and heatstroke-related ambulance dispatch (HSAD) cases in Japan using daily national data.

**Methods:**

Daily HSAD counts and meteorological data (daily maximum temperature (°C), relative humidity (%), wind speed (m/s), and sunshine duration (h)) were analyzed for June–September (summer), 2015–2019, across 47 Japanese prefectures. A time-stratified case-crossover study with conditional quasi-Poisson regression combined with a distributed lag non-linear model was used to estimate maximum temperature-HSAD associations in Japan between 2015 and 2019. A random-effects meta-analysis pooled country-level association.

**Results:**

A total of 300,528 HSAD cases were analyzed. Higher temperatures were associated with an increased risk of HSAD cases. The overall lag-cumulative relative risk at the 95th percentile of maximum temperature (35.3 °C) compared to the minimum morbidity temperature (18.2 °C) was 19.23, with a 95 % confidence interval of 13.95 to 26.51. Lagged effects of higher temperatures persisted for up to 2 days. Significant geographical variation in HSAD cases was observed (Cochran *Q* test, *p* < 0.001; *I*^2^ = 84.0 %). The association between maximum temperature and HSAD cases was significantly modified by the use of air conditioning (likelihood ratio test, *p* = 0.01).

**Conclusions:**

Our study demonstrated the influence of heat on HSAD cases in Japan, emphasizing the necessity of preventive strategies to reduce the impact of temperature on heatstroke morbidity.

## Highlights

• Temperature was associated with heatstroke-related ambulance dispatches in Japan.
• Risk persisted for approximately 2 days post-exposure.
• Geographical heterogeneity among prefectures was observed.
• Air conditioning use modified the exposure–response association.

## 1 Introduction

Climate change has markedly increased the frequency, duration, and intensity of heat waves, raising substantial societal concerns (Luber and McGeehin, 2008). This phenomenon has prompted a surge in epidemiological studies investigating the associations between ambient temperature and mortality (i.e., death counts) worldwide(Wu et al., 2022). These studies consistently demonstrate that temperature-mortality relationships vary across regions, with differing impacts of heat and cold, and variations in the shape of the temperature-mortality curve (Wu et al., 2022). However, most studies have focused on mortality as health outcome, representing a limited subset of severe cases at the top of the health impact pyramid. Indeed, the short-term effects of ambient temperature on morbidity (e.g., heatstroke incidence) remain underexplored in multi-location analyses. Furthermore, few studies have quantified nationwide spatial variations in this association. This study aimed to investigate heatstroke incidence patterns in Japan using data from a 5-year follow-up period across all prefectures and to investigate the temperature-morbidity association on a national scale.

## 2 Materials and methods

### 2.1 Data collection

We collected daily heatstroke-related ambulance dispatches (HSAD) count (International Classification of Diseases 10 [ICD10]: codes T67.0-T67.3 and T67.5-T67.8) for the summer months (June–September) from 2015 to 2019 across all 47 Japanese prefectures using the Fire and Disaster Management Agency database, managed by the Ministry of Internal Affairs (FDMA, 2024; Hatakeyama et al., 2021; Hatakeyama and Seposo, 2022; Seposo et al., 2021). Meteorological variables such as daily maximum temperature (°C), relative humidity (%), wind speed (m/s), and sunshine duration (h) were obtained from a single monitoring station operated by the Japan Meteorological Agency in each of the 47 Japanese prefectures (JMA, 2024). Data were anonymized, aggregated, derived from publicly accessible databases, and exempt from ethical approval.

### 2.2 Statistical analyses

A two-stage design was conducted (Sera and Gasparrini, 2022). In the first stage, a time-stratified case-crossover design was applied to model prefectural data using a conditional quasi-Poisson regression combined with a distributed lag non-linear model (DLNM) (Tobias et al., 2024). This statistical design controls for fixed individual characteristics, such as sex, education, lifestyle, and chronic diseases, within the small-time window (Tobias et al., 2024). Time stratification, defined through a three-way interaction of year, month, and day of the week, accounted for seasonality, long-term trends, and day-of-week effects. The DLNM's cross-basis function captured non-linear and delayed effects, employing a natural cubic spline for the exposure-response relationship with knots at the 25th, 50th, and 75th percentiles of maximum temperature. Another natural cubic spline was used for the lag-response relationship over a 7-day lag period, with three equally spaced knots on a log scale (Oka et al., 2023). The model also adjusted for confounders such as relative humidity, wind speed, and sunshine duration, each modeled with a natural cubic spline with three degrees of freedom and considered national public holidays (Hatakeyama et al., 2021; Hatakeyama and Seposo, 2022; Seposo et al., 2021). In the second stage, a multivariate meta-analysis using a random-effects model with maximum likelihood estimation (intercept-only) was performed to pool the prefecture-specific estimates from the first stage (Gasparrini and Armstrong, 2013). Annual prevalence of air conditioning (AC) in households with at least two occupants of each prefecture was incorporated as a meta-predictor to examine potential spatial variations (e-Stat, 2024). The modifying effect of AC use was assessed by predicting temperature-morbidity relationships at the 10th and 90th percentiles of the AC distribution. Residual heterogeneity was assessed using *I*^2^ statistics and Cochran's *Q* test, while the statistical significance of this effect was evaluated through a likelihood ratio test. Relative risks (RRs) and 95 % confidence intervals (CIs) were estimated using the minimum risk temperature percentile of the temperature distribution of each prefecture and at the country level, defined as the minimum of the exposure-response curve, as the reference value. Several sensitivity analyses were performed to evaluate the robustness of the findings. First, the knot placements were adjusted from the 25th, 50th, and 75th percentiles to the 10th, 50th, and 90th percentiles, as well as the 10th, 75th, and 90th percentiles. Second, the maximum lag period was extended from 7 to 14 days. All statistical computations were conducted using R software (version 4.1.0) using the packages *dlnm* and *mixmeta*.

### 2.3 Ethical considerations

This study was based exclusively on publicly available, aggregated datasets that had been de-identified and fully anonymized prior to analysis. Secondary analyses of such anonymized, aggregate data are not classified as human-subjects research. Consequently, approval by an institutional ethics committee was not required, and the requirement to obtain individual informed consent was waived. The study was conducted in accordance with the Declaration of Helsinki.

## 3 Results

**Fig. 1:**
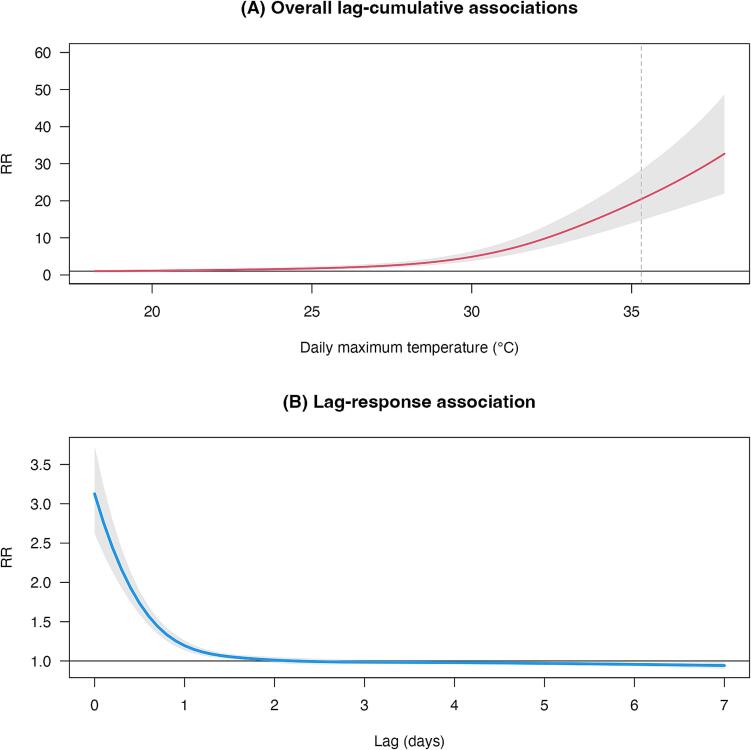
Association curves between daily maximum temperature and daily heatstroke-related ambulance dispatch counts among residents of all ages across 47 Japanese prefectures during summer (June–September) 2015–2019. (A) Overall lag-cumulative associations between maximum temperature and HSAD (red line). The shaded gray area represents the 95 % CI. Vertical lines denote RR calculations for heat based on the 95th percentile of maximum temperature (i.e., 35.3 °C). The maximum temperature reference value is represented by the minimum morbidity temperature, calculated as 18.2 °C. (B) Lag-response association between maximum temperature and HSAD (blue line) for extreme high daily maximum temperatures (95th percentile). Shaded gray area is the 95 % CI. RR: relative risk, CI: confidence interval, HSAD: heatstroke-related ambulance dispatches. (For interpretation of the references to colour in this figure legend, the reader is referred to the web version of this article.)

**Fig. 2:**
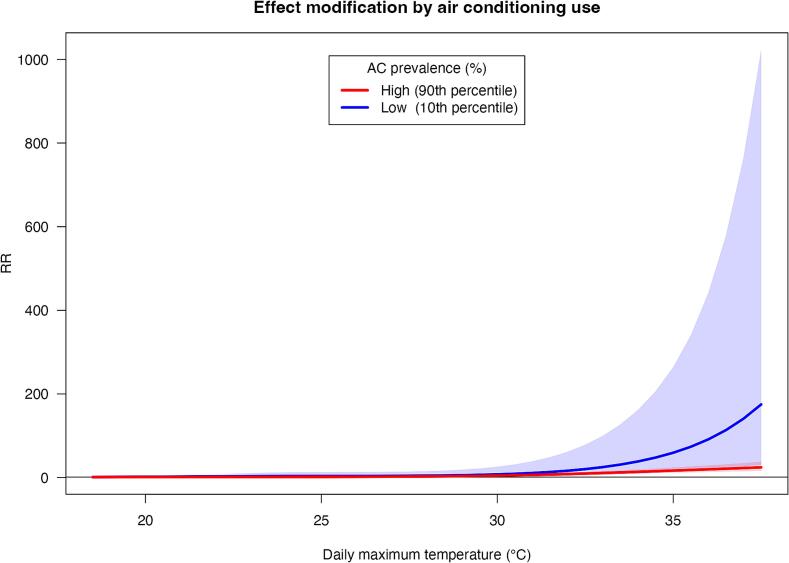
Associations between daily maximum temperature and daily heatstroke-related ambulance dispatch counts among residents of all ages across 47 Japanese prefectures during summer (June–September) 2015–2019, stratified by household air-conditioning prevalence. Predictions for the 10th (blue line) and 90th (red line) percentiles of AC use were derived using univariate meta-regression. Shaded regions surrounding the lines represent 95 % CIs. The maximum temperature reference value is represented by the minimum morbidity temperature, calculated as 18.2 °C. RR: relative risk, CI: confidence interval, HSAD: heatstroke-related ambulance dispatches; AC, air conditioning. (For interpretation of the references to colour in this figure legend, the reader is referred to the web version of this article.)

In total, 300,528 HSAD cases were analyzed between 2015 and 2019 (Table S1). The pooled median maximum temperature was 29.3 °C, with the 5th and 95th percentiles at 22.7 °C and 35.3 °C, respectively. Fig. 1A depicts the overall lag-cumulative association between maximum temperature and HSAD cases across Japan, revealing an exponential relationship nationwide. More specifically, exposure to extreme high daily maximum temperatures (35.3 °C at the 95th percentile) was associated with an approximate twentyfold increase in HSAD risk cases compared to the exposure to the reference temperature (RR = 19.23, 95 % CI = 13.95, 26.51). Fig. 1B illustrates the lag-response curve, highlighting a delayed effect of high maximum temperatures on HSAD cases, with a notable increase in risk occurring approximately 1–2 days after exposure. A multivariate random-effects meta-analysis (intercept-only) identified substantial geographical heterogeneity in HSAD cases (Cochran *Q* test, *p* < 0.001; *I*^2^ = 84.0 %). The meta-regression analysis, incorporating AC prevalence as a meta-predictor, demonstrated a marked difference in the overall cumulative associations observed (Cochran *Q* test, *p* < 0.001; *I*^2^ = 83.1 %; likelihood ratio test, *p* = 0.01) (Fig. 2). Exposure-response and lag-specific associations for all 47 Japanese prefectures are presented in Figs. S1 and S2. Sensitivity analyses confirmed the robustness of the findings across various parameter adjustments, as shown in Fig. S3.

## 4 Discussion

This nationwide time-series study found an exponential association between daily maximum temperature and HSAD cases across Japan. The study's strength lies in using multi-location analysis, accounting for seasonality, long-term trends, and potential confounding factors. Our results aligned with those of a meta-analysis (RR = 1.35; 95 % CI = 1.29, 1.41) from a recent systematic review on the association between high temperature and heatstroke mortality (Faurie et al., 2022). Furthermore, other studies conducted in Japan have consistently reported positive associations between heatstroke incidence and environmental stressors, such as maximum temperature, wet-bulb globe temperature, and the Universal Thermal Climate Index (Hatakeyama et al., 2021; Hatakeyama and Seposo, 2022; Iwamoto and Ohashi, 2021; Oka et al., 2023; Seposo et al., 2021; Ueno et al., 2021).

The highest HSAD risk occurred on the day of exposure (i.e., lag 0 days) and remained elevated for more than 2 days. Heatstroke, despite its rapid progression and a high case-mortality rate, often develops gradually over several days (Tobías et al., 2023). Supporting these findings, studies in Spain found heat-related increases in all-cause mortality persisting for up to 5 days (Tobías et al., 2014). Likewise, a recent epidemiological study in Japan investigating the relationship between wet-bulb globe temperature and heatstroke incidence observed delayed effects of heat exposure lasting 0–2 days (Oka et al., 2023). Collectively, these findings indicated that the risk of emergency transport for acute heatstroke is highest within a few days following heat exposure.

This study demonstrated significant spatial heterogeneity in HSAD risks across prefectures, indicating the presence of regional disparities in Japan (i.e., *I*^2^ = 84.0 %). Meta-regression analysis revealed AC's modifying effects. In prefectures with low AC utilization, the risk function was markedly elevated at higher temperatures and exhibited a significantly steeper gradient than in those with greater AC penetration. Indeed, physiological heat acclimatization to local meteorological contributes to regional differences in heatstroke incidence. Behavioral adaptations, such as increased AC use, are also key factors influencing regional disparities (Chua et al., 2023; Oka et al., 2023). Additionally, this modifying effect may stem from variations in the epidemiological characteristics of distinct population subgroups. These factors likely contributed to the observed spatial variations in HSAD risks, warranting further research that incorporates diverse socio-economic and demographic variables across different regions in Japan. Although not all factors modifying the decline in incidence were identified, the findings suggested that preventive strategies should prioritize preparing local populations and targeted adaptation measures to effectively address future temperature changes.

A major strength of this study was the availability of long-term data from all 47 Japanese prefectures, facilitating a comprehensive nationwide assessment. However, our study had some limitations. First, the study was limited to Japan, which may constrain the generalizability of the findings to other regions or countries. Lastly, the lack of individual-level data prevented stratified analyses by age, sex, or severity, hindering a thorough evaluation of subgroup vulnerabilities.

## 5 Conclusions

In conclusion, elevated summer temperatures are significantly associated with increased HSAD cases in Japan. These findings highlight the importance of developing prevention strategies to address climate change-induced rises, as summer temperatures could substantially increase the risk of heatstroke morbidity.

## CRediT authorship contribution statement

**Keita Wagatsuma:** Writing – original draft, Supervision, Project administration, Methodology, Funding acquisition, Data curation, Conceptualization.

## Funding

This study was supported by a Grant-in-Aid for Scientific Research (KAKENHI) from the 10.13039/501100001691Japan Society for the Promotion of Science (Grant Nos. 24K23680 and 25K20622). The funding agency played no role in the study design, data collection, data analysis, data interpretation, or manuscript writing.

## Declaration of competing interest

The authors declare that they have no known competing financial interests or personal relationships that could have appeared to influence the work reported in this paper.

## Data Availability

Data will be made available on request.
