# Supplementary material for: Effects of high summer temperatures on heatstroke-related ambulance dispatches in Japan: A nationwide time-stratified case-crossover analysis
Source: Prev Med Rep. 2025 Jun 9;55:103134. doi: 10.1016/j.pmedr.2025.103134 (PMC12182763; doi:10.1016/j.pmedr.2025.103134)
Supplement: Supplementary material [file mmc1.docx]

**Table S1. Descriptive statistics of daily heatstroke‐related ambulance dispatch counts among residents of all ages, meteorological variables, and household air‐conditioning prevalence in 47 Japanese prefectures during summer (June–September) 2015–2019.**

| **Prefecture** | **AC use (%)** | **Daily observed HSAD cases** | | **Maximum temperature (°C)** | **Relative humidity (%)** | **Wind speed (m/s)** | **Sunshine duration (h)** |
| --- | --- | --- | --- | --- | --- | --- | --- |
|  |  | **N** | **Median (5^th^, 95^th^)** | **Median (5^th^, 95^th^)** | **Median (5^th^, 95^th^)** | **Median (5^th^, 95^th^)** | **Median (5^th^, 95^th^)** |
| Aichi | 97.2 | 19,487 | 11.0 (0.0, 122.0) | 30.4 (23.8, 36.6) | 70.0 (51.0, 90.0) | 2.7 (1.8, 5.0) | 5.5 (0.0, 12.8) |
| Akita | 78.9 | 2,247 | 1.0 (0.0, 18.0) | 26.6 (19.9, 33.5) | 76.0 (60.0, 92.0) | 3.2 (2.2, 6.2) | 6.2 (0.0, 13.2) |
| Aomori | 56.4 | 1,819 | 1.0 (0.0, 15.0) | 25.6 (19.1, 32.1) | 77.2 (65.4, 91.1) | 3.0 (1.8, 5.9) | 5.9 (0.0, 12.9) |
| Chiba | 95.1 | 13,088 | 7.0 (0.0, 94.0) | 28.8 (22.6, 34.0) | 77.0 (61.0, 92.0) | 3.5 (2.2, 7.4) | 4.5 (0.0, 12.4) |
| Ehime | 96.6 | 4,172 | 3.0 (0.0, 26.0) | 30.1 (23.9, 35.0) | 72.0 (57.0, 91.0) | 2.0 (1.3, 2.8) | 5.5 (0.0, 12.4) |
| Fukui | 97.8 | 1,863 | 1.0 (0.0, 13.0) | 29.5 (22.8, 35.8) | 75.5 (60.0, 90.0) | 2.5 (1.6, 4.4) | 5.6 (0.0, 12.6) |
| Fukuoka | 95.5 | 12,118 | 8.0 (0.0, 75.0) | 29.7 (24.4, 35.8) | 76.0 (63.0, 92.0) | 2.5 (1.6, 4.8) | 6.3 (0.0, 12.5) |
| Fukushima | 75.0 | 5,354 | 3.0 (0.0, 41.0) | 28.2 (20.6, 36.4) | 76.0 (58.0, 94.0) | 2.1 (1.3, 3.5) | 3.5 (0.0, 12.1) |
| Gifu | 92.5 | 5,945 | 4.0 (0.0, 38.0) | 30.4 (23.8, 37.3) | 69.0 (51.0, 90.0) | 2.5 (1.5, 4.6) | 6.0 (0.0, 13.0) |
| Gunma | 90.3 | 5,927 | 3.0 (0.0, 41.0) | 29.1 (21.4, 36.8) | 72.0 (52.0, 93.0) | 2.0 (1.2, 3.3) | 4.5 (0.0, 12.4) |
| Hiroshima | 95.9 | 7,930 | 6.0 (0.0, 46.0) | 29.7 (23.9, 35.9) | 66.0 (51.0, 85.0) | 2.9 (1.8, 4.9) | 6.0 (0.0, 12.4) |
| Hokkaido | 26.6 | 5,220 | 2.0 (0.0, 40.0) | 24.4 (16.9, 30.8) | 73.0 (58.0, 88.0) | 3.0 (1.8, 6.1) | 5.8 (0.0, 13.2) |
| Hyogo | 96.8 | 14,683 | 10.0 (0.0, 92.2) | 29.4 (24.3, 35.2) | 72.0 (55.0, 88.0) | 3.3 (2.2, 7.0) | 6.6 (0.0, 12.6) |
| Ibaraki | 94.9 | 7,312 | 4.0 (0.0, 56.0) | 27.6 (21.3, 34.6) | 81.0 (67.0, 93.0) | 2.2 (1.5, 4.4) | 4.5 (0.0, 12.0) |
| Ishikawa | 96.0 | 2,782 | 2.0 (0.0, 20.0) | 28.9 (22.0, 34.6) | 72.8 (60.1, 86.9) | 3.1 (2.0, 5.7) | 6.4 (0.0, 13.0) |
| Iwate | 61.2 | 2,496 | 1.0 (0.0, 20.0) | 26.4 (19.1, 33.5) | 78.0 (64.0, 95.0) | 2.8 (1.8, 4.4) | 4.6 (0.0, 12.1) |
| Kagawa | 98.0 | 2,971 | 2.0 (0.0, 20.0) | 30.3 (24.2, 36.0) | 72.0 (57.0, 90.0) | 2.0 (1.5, 4.2) | 6.2 (0.0, 12.5) |
| Kagoshima | 95.0 | 6,186 | 6.0 (0.0, 33.0) | 30.9 (24.5, 35.2) | 78.0 (66.0, 94.0) | 2.7 (1.8, 5.4) | 5.8 (0.0, 11.8) |
| Kanagawa | 94.8 | 14,160 | 8.0 (0.0, 102.1) | 28.7 (21.8, 34.3) | 80.1 (64.9, 97.3) | 3.0 (2.0, 6.1) | 5.1 (0.0, 12.7) |
| Kochi | 90.8 | 2,465 | 2.0 (0.0, 16.0) | 30.1 (24.6, 34.5) | 79.0 (62.0, 95.0) | 1.7 (1.1, 2.4) | 5.2 (0.0, 12.0) |
| Kumamoto | 96.3 | 6,661 | 5.0 (0.0, 39.0) | 30.6 (24.3, 36.5) | 76.0 (59.0, 95.0) | 2.1 (1.3, 4.3) | 6.0 (0.0, 12.1) |
| Kyoto | 97.7 | 8,052 | 6.0 (0.0, 47.1) | 30.9 (24.1, 36.9) | 68.5 (57.7, 84.9) | 2.0 (1.5, 3.5) | 4.6 (0.0, 11.6) |
| Mie | 98.0 | 5,359 | 3.0 (0.0, 36.1) | 28.9 (22.9, 34.6) | 71.0 (52.0, 87.0) | 2.9 (1.7, 5.9) | 6.0 (0.0, 12.8) |
| Miyagi | 75.0 | 4,634 | 2.0 (0.0, 37.0) | 26.2 (19.9, 33.6) | 80.0 (58.0, 95.0) | 2.6 (1.8, 4.4) | 3.8 (0.0, 12.8) |
| Miyazaki | 92.4 | 3,575 | 3.0 (0.0, 20.0) | 29.8 (23.7, 34.4) | 83.0 (68.0, 96.0) | 2.7 (1.6, 5.5) | 4.6 (0.0, 12.1) |
| Nagano | 64.1 | 4,199 | 2.0 (0.0, 29.0) | 28.4 (20.9, 35.1) | 75.0 (61.0, 91.0) | 2.3 (1.4, 4.3) | 5.7 (0.0, 11.9) |
| Nagasaki | 95.8 | 3,947 | 2.0 (0.0, 26.0) | 29.5 (23.7, 34.7) | 81.0 (64.0, 95.0) | 2.0 (1.2, 4.2) | 6.2 (0.0, 11.9) |
| Nara | 96.9 | 4,369 | 3.0 (0.0, 25.0) | 30.3 (23.6, 36.3) | 74.0 (61.0, 93.0) | 1.6 (0.9, 3.3) | 5.2 (0.0, 11.8) |
| Niigata | 93.9 | 5,836 | 3.0 (0.0, 45.0) | 27.5 (21.4, 34.3) | 76.0 (62.0, 91.0) | 2.3 (1.6, 5.1) | 5.9 (0.0, 13.1) |
| Oita | 94.6 | 3,572 | 3.0 (0.0, 23.0) | 29.4 (23.2, 35.1) | 77.0 (62.0, 95.0) | 2.3 (1.4, 4.0) | 5.3 (0.0, 12.4) |
| Okayama | 95.4 | 7,132 | 5.0 (0.0, 44.0) | 30.2 (24.2, 36.2) | 74.0 (59.0, 91.0) | 2.6 (1.9, 4.9) | 5.8 (0.0, 12.2) |
| Okinawa | 88.9 | 4,455 | 6.0 (0.0, 17.0) | 31.6 (27.9, 33.7) | 79.0 (69.0, 91.0) | 4.9 (2.5, 9.0) | 6.7 (0.0, 11.4) |
| Osaka | 97.7 | 21,815 | 14.5 (1.0, 137.0) | 30.8 (24.4, 36.5) | 69.0 (55.0, 87.0) | 2.3 (1.6, 4.0) | 6.2 (0.0, 12.1) |
| Saga | 97.7 | 2,764 | 2.0 (0.0, 17.0) | 30.5 (24.3, 36.6) | 74.0 (59.0, 93.0) | 2.8 (1.7, 5.9) | 6.0 (0.0, 12.2) |
| Saitama | 96.4 | 18,095 | 10.0 (0.0, 138.0) | 29.4 (21.9, 37.4) | 74.0 (55.0, 94.0) | 2.1 (1.4, 3.4) | 4.5 (0.0, 12.2) |
| Shiga | 96.4 | 3,458 | 2.0 (0.0, 22.0) | 29.1 (22.7, 35.2) | 74.0 (62.0, 90.0) | 2.2 (1.5, 5.2) | 6.2 (0.0, 12.8) |
| Shimane | 94.0 | 1,992 | 1.0 (0.0, 13.0) | 28.6 (22.2, 35.5) | 80.0 (65.0, 93.0) | 2.7 (1.7, 5.7) | 5.5 (0.0, 12.7) |
| Shizuoka | 93.5 | 7,782 | 5.0 (0.0, 53.1) | 29.4 (23.4, 34.5) | 76.0 (61.0, 90.0) | 2.0 (1.4, 3.3) | 5.3 (0.0, 11.8) |
| Tochigi | 93.7 | 4,523 | 2.0 (0.0, 33.0) | 28.5 (21.5, 35.4) | 79.0 (62.0, 94.0) | 2.7 (1.8, 5.0) | 3.2 (0.0, 11.2) |
| Tokushima | 97.3 | 2,189 | 2.0 (0.0, 14.0) | 29.7 (23.7, 35.0) | 77.0 (60.0, 93.0) | 2.7 (1.7, 5.2) | 6.3 (0.0, 12.6) |
| Tokyo | 93.0 | 22,886 | 11.0 (0.0, 184.1) | 29.0 (21.9, 35.1) | 79.0 (63.0, 98.0) | 2.7 (1.8, 5.0) | 3.9 (0.0, 12.2) |
| Tottori | 91.5 | 1,881 | 1.0 (0.0, 13.0) | 29.6 (22.5, 36.3) | 77.0 (62.0, 91.0) | 2.5 (1.7, 4.3) | 5.7 (0.0, 12.7) |
| Toyama | 94.8 | 2,033 | 1.0 (0.0, 16.0) | 28.5 (22.2, 35.2) | 76.0 (61.0, 94.0) | 2.4 (1.6, 4.4) | 5.4 (0.0, 12.5) |
| Wakayama | 98.2 | 3,256 | 3.0 (0.0, 20.0) | 30.4 (24.4, 35.4) | 74.0 (58.0, 89.0) | 3.2 (2.1, 6.0) | 6.9 (0.0, 12.8) |
| Yamagata | 84.0 | 2,447 | 1.0 (0.0, 18.0) | 28.1 (21.0, 35.5) | 74.0 (58.0, 91.0) | 1.7 (1.1, 3.1) | 4.8 (0.0, 12.1) |
| Yamaguchi | 94.1 | 3,300 | 2.0 (0.0, 21.0) | 30.1 (24.2, 37.0) | 77.0 (64.0, 93.0) | 1.8 (1.1, 3.3) | 5.4 (0.0, 12.4) |
| Yamanashi | 80.8 | 2,091 | 1.0 (0.0, 15.0) | 30.7 (23.2, 36.9) | 68.0 (51.0, 88.0) | 2.1 (1.2, 3.1) | 6.0 (0.0, 11.7) |
| Nationwide | 95.0 (62.0, 98.0) | 300,528 | 4.0 (0.0, 43.2) ^a^ | 29.3 (22.7, 35.3) ^b^ | 75.2 (58.8, 91.7) ^c^ | 2.5 (1.6, 4.8) ^d^ | 5.5 (0.0, 12.4) ^e^ |

HSAD: heatstroke-related ambulance dispatches, AC: air conditioning, NA: not available.

^a^ Median (5^th^ and 95^th^ percentile) of prefecture-specific annual air conditioning use distributions over the 5-year study period.

^b^ Mean of medians (5^th^ and 95^th^ percentile) of prefecture-specific daily heatstroke-related ambulance dispatch distributions over the 5-year study period.

^c^ Mean of medians (5^th^ and 95^th^ percentile) of prefecture-specific daily maximum temperature distributions over the 5-year study period.

^d^ Mean of medians (5^th^ and 95^th^ percentile) of prefecture-specific daily relative humidity distributions over the 5-year study period.

^e^ Mean of medians (5^th^ and 95^th^ percentile) of prefecture-specific daily wind speed distributions over the 5-year study period.

^f^ Mean of medians (5^th^ and 95^th^ percentile) of prefecture-specific daily sunshine duration distributions over the 5-year study period.

**
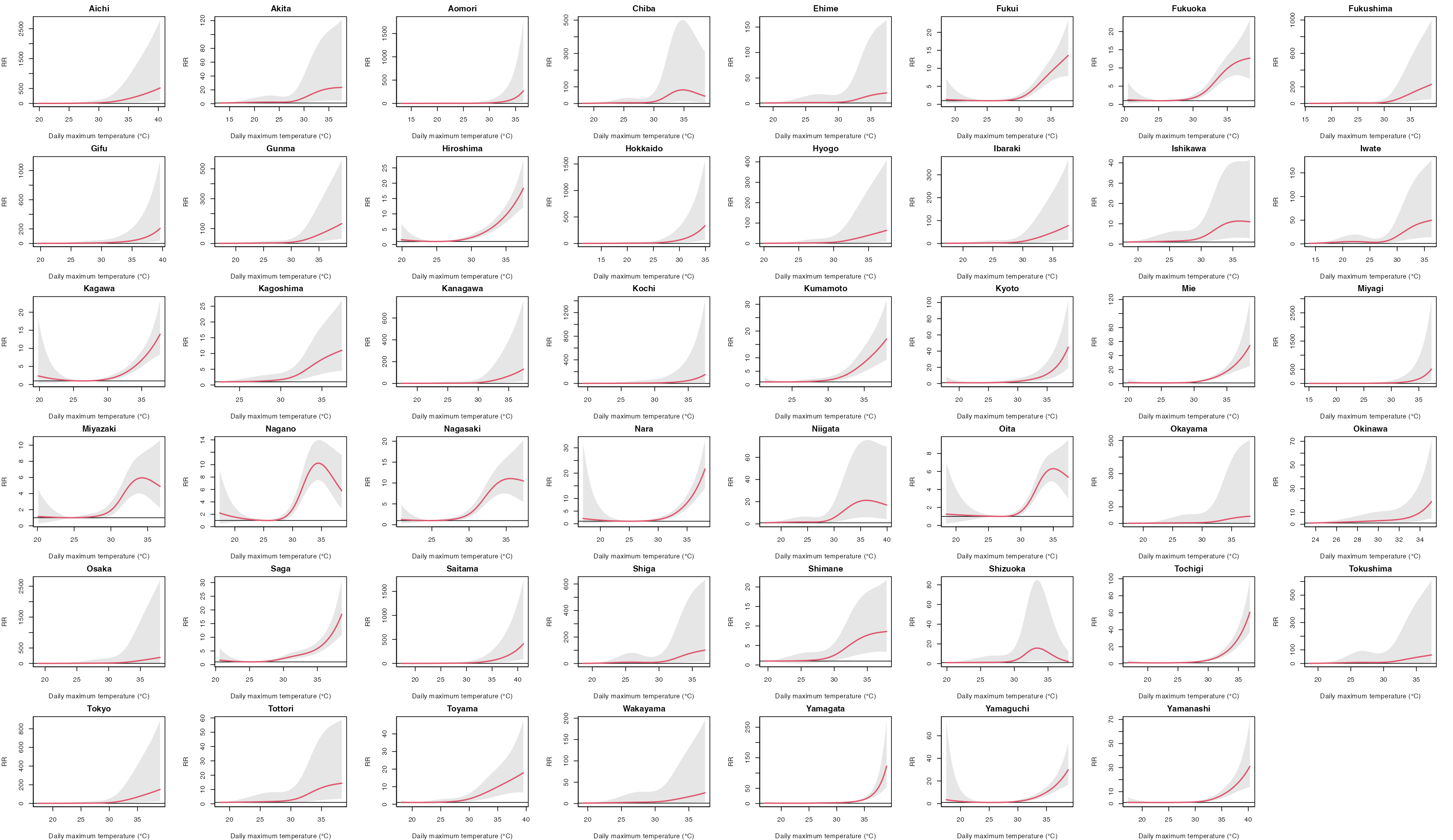
**

**Figure S1. Associations between daily maximum temperature and daily heatstroke‐related ambulance dispatch counts among residents of all ages in each of the 47 Japanese prefectures during summer (June–September) 2015–2019.** RR: relative risk, HSAD: heatstroke-related ambulance dispatches.

**
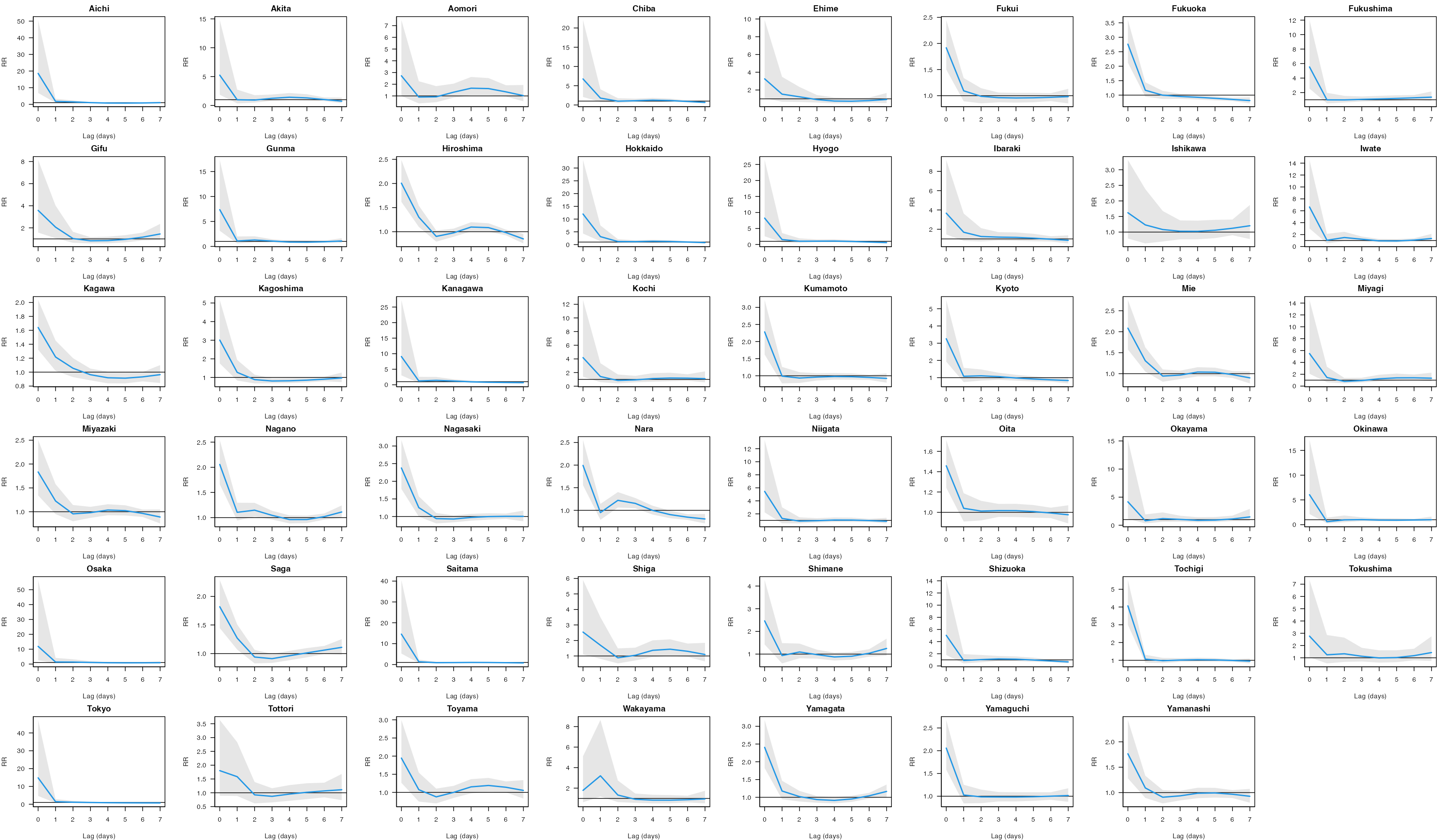
**

**Figure S2. Lag‐response associations between daily maximum temperature and daily heatstroke‐related ambulance dispatch counts among residents of all ages in each of the 47 Japanese prefectures during summer (June–September) 2015–2019.** RR: relative risk, HSAD: heatstroke-related ambulance dispatches.

**
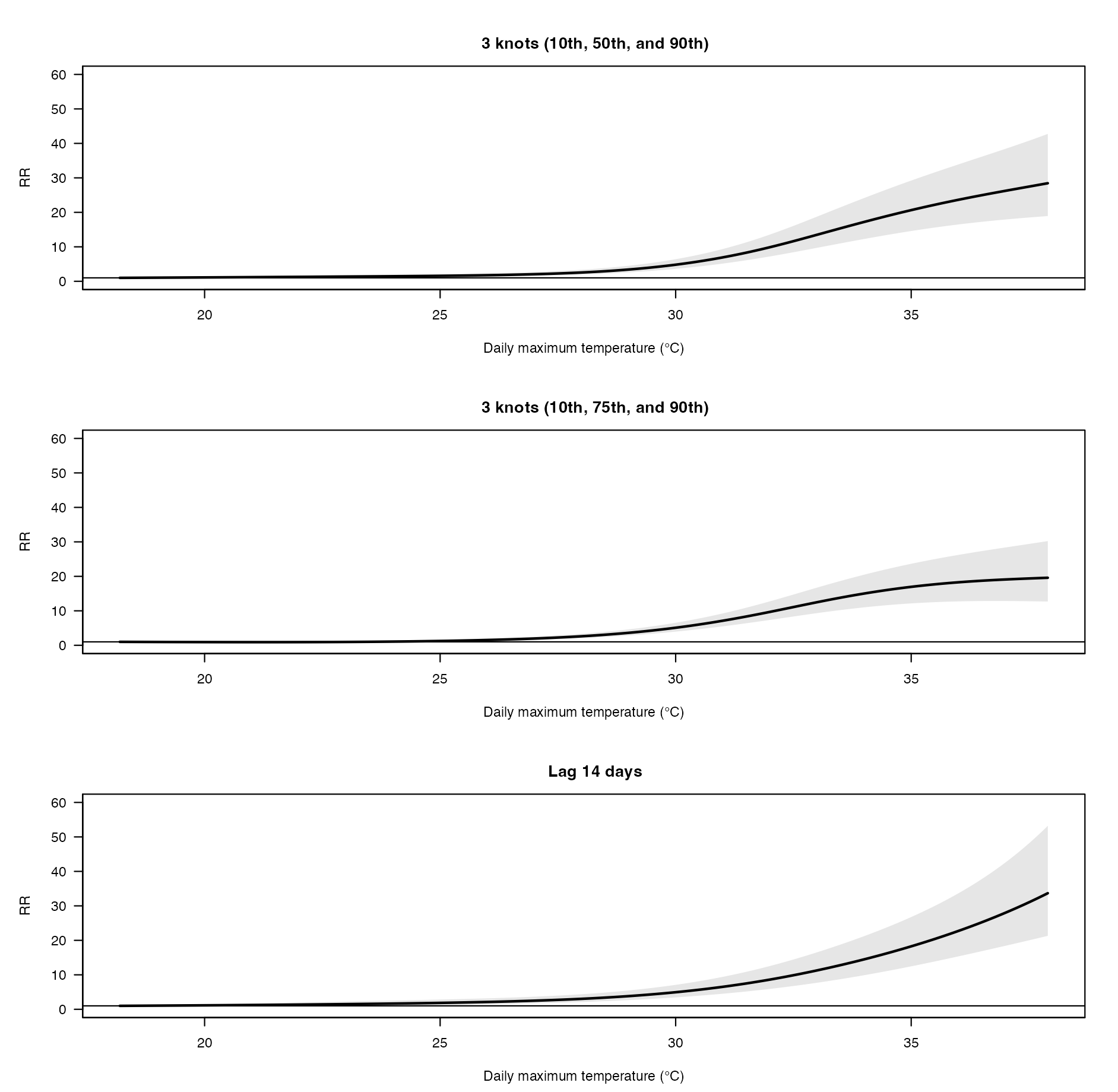
**

**Figure S3. Sensitivity analyses of associations between daily maximum temperature and daily heatstroke‐related ambulance dispatch counts among residents of all ages across 47 Japanese prefectures during summer (June–September) 2015–2019.** RR: relative risk, HSAD: heatstroke-related ambulance dispatches.
